# Supplementary material for: The mitochondrial genome of Paragonimus westermani (Kerbert, 1878), the Indian isolate of the lung fluke representative of the family Paragonimidae (Trematoda)
Source: PeerJ. 2014 Aug 12;2:e484. doi: 10.7717/peerj.484 (PMC4137670; doi:10.7717/peerj.484)
Supplement: File S1 [file peerj-02-484-s001.pdf]

SO_1994:

Gel pic 1: (set 1)

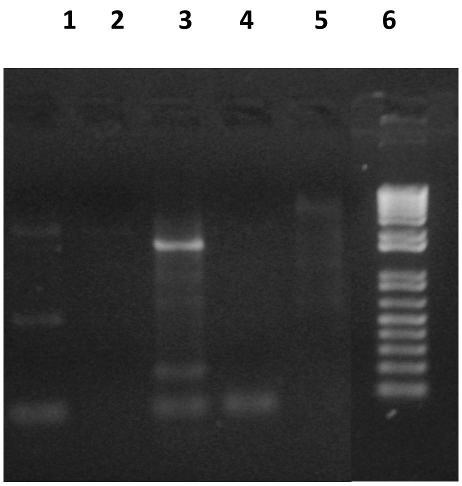

LANE	SAMPLE
2	2F+2R
3	3F+3R
4	4F+4R
5	Nill
6	6F+6R
L	1 KB Plus Ladder

Primer sequences:

2_F	TGAGAGGTATCTTCAGGCGGA
2_R	ACAACGTGTAAACCTCGCAGAA
3_F	TTCTCTTTCTGTGGCTTGCTTATG
3_R	AGATAAAGCAGAACATCACCGGA
4_F	AAAGTGGTTTGTGTGCTGTCCTT
4_R	AACTACCATGTCGCGACCG
6_F	TGCTGCGATCACCATGCT
6_R	ATGAGACCTCTCCTCGCCTCT

Gel pic 2: (set 3)

1 2 3 4

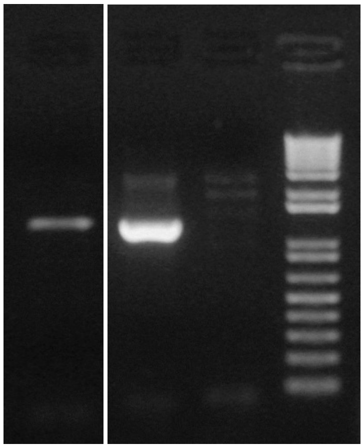

LANE	SAMPLE
1	1 KB Plus Ladder
2	3F+3R
3	9F+9R
4	Nill
5	1kb+ ladder

Primer sequences:

3F	ATGGTGTTGGATGTTGAGAGG
3R	ACCAACGCTTCCCATCTAACT
9F	TGAGGGGTTTTCCTACTGCTT
9R	AAAAGCTGCAACAAACAAAGC

Gel pic 3: (set2)

1 2 3 4 5 6 7 8

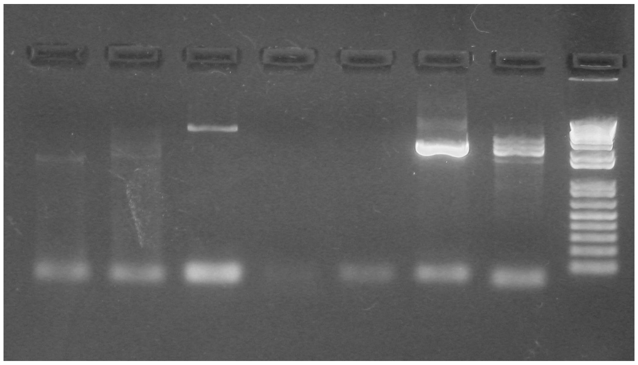

LANE	SAMPLE
1	2F+3R
2	3F+4R
3	5F+6R
4	Nill
5	Nill
6	7F+8R
7	8F+9R
8	1Kb+ Ladder

Primer sequences:

2F	ACAATTCCTGGATGTTTCTCGT
3R	ACCAACGCTTCCCATCTAACT
3F	ATGGTGTTGGATGTTGAGAGG
4R	TCACACGACACCCTTTCCTTA
5F	TTAAGGAAAGGGTGTCGTGTG
6R	GGCCCACTGATGTAAATCCT
7F	CTCGTGTTTAGTGGGTGTCGT
8R	AAGCAGTAGGAAAACCCCTCA
8F	CGTTTGGTTCAGATGCTTTGT
9R	AAAAGCTGCAACAAACAAAGC

Gel pic 4: (set 3)

1 2 3

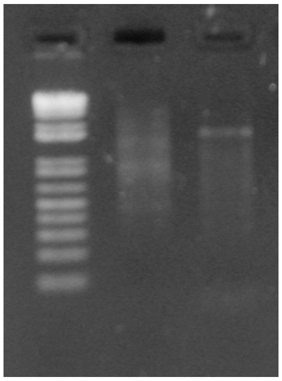

Lane	Sample
1	1kb+ Ladder
2	Nill
3	3F+4R

Primer sequences:

3_F	TGGTGATGCTCGCTTGTGAC
4_R	ACTACACCCATTGCCTCGG

Gel pic 5:(set3)

1 2

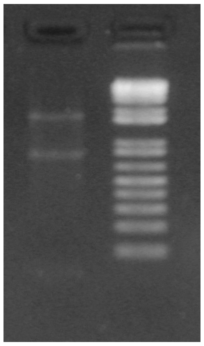

Lane	Sample
1	6F+6R
2	1kb+ Ladder

Primer sequences:

PWG6_F	CTGGCGGCTCTTTCTGTAG
PWG6_R	ACGAACAACCACACATAATCC

Gel pic 6:(set3)

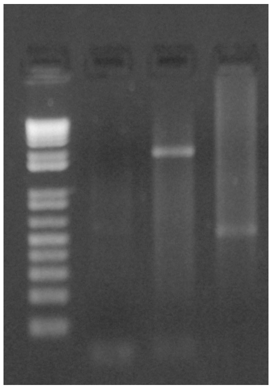

Lane	Sample
1	1kb+ Ladder
2	Nill
3	2F+3R
4	3F+3R

Primer sequences:

3_F	TGGTGATGCTCGCTTGTGAC
3_R	CGAGCACTGTCACTGTTGG
2_F	AGGTCAATGTGCTGCTTACG
3_R	CGAGCACTGTCACTGTTGG

Gel pic 7:(set3)

1 2

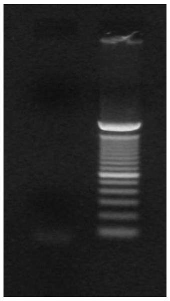

Lane	Sample
1	7F+7R
2	1kb+ Ladder

Primer sequences:

7_F	CTTTAGAATGGGTGATGGTTG
7_R	CTACAGAAAGAGCCGCCAG
